# Role of Lactobacilli and Lactoferrin in the Mucosal Cervicovaginal Defense

**DOI:** 10.3389/fimmu.2018.00376

**Published:** 2018-03-01

**Authors:** Piera Valenti, Luigi Rosa, Daniela Capobianco, Maria Stefania Lepanto, Elisa Schiavi, Antimo Cutone, Rosalba Paesano, Paola Mastromarino

**Affiliations:** ^1^Department of Public Health and Infectious Diseases, University of Rome La Sapienza, Rome, Italy; ^2^Department of Gynecological-Obstetric and Urological Sciences, University of Rome La Sapienza, Rome, Italy

**Keywords:** lactobacilli, lactoferrin, cervicovaginal defense, vaginal homeostasis, inflammation

## Abstract

The innate defense system of the female mucosal genital tract involves a close and complex interaction among the healthy vaginal microbiota, different cells, and various proteins that protect the host from pathogens. Vaginal lactobacilli and lactoferrin represent two essential actors in the vaginal environment. Lactobacilli represent the dominant bacterial species able to prevent facultative and obligate anaerobes outnumber in vaginal microbiota maintaining healthy microbial homeostasis. Several mechanisms underlie the protection exerted by lactobacilli: competition for nutrients and tissue adherence, reduction of the vaginal pH, modulation of immunity, and production of bioactive compounds. Among bioactive factors of cervicovaginal mucosa, lactoferrin, an iron-binding cationic glycoprotein, is a multifunctional glycoprotein with antibacterial, antifungal, antiviral, and antiparasitic activities, recently emerging as an important modulator of inflammation. Lactobacilli and lactoferrin are largely under the influence of female hormones and of paracrine production of various cytokines. Lactoferrin is strongly increased in lower genital tract mucosal fluid of women affected by *Neisseria gonorrheae, Chlamydia trachomatis*, and *Trichomonas vaginalis* infections promoting both innate and adaptive immune responses. In vaginal dysbiosis characterized by low amounts of vaginal lactobacilli and increased levels of endogenous anaerobic bacteria, the increase in lactoferrin could act as an immune modulator assuming the role normally played by the healthy microbiota in vaginal mucosa. Then lactoferrin and lactobacilli may be considered as biomarkers of altered microbial homeostasis at vaginal level. Considering the shortage of effective treatments to counteract recurrent and/or antibiotic-resistant bacterial infections, the intravaginal administration of lactobacilli and lactoferrin could be a novel efficient therapeutic strategy and a valuable tool to restore mucosal immune homeostasis.

## Introduction

The basic structures of the female reproductive system are ovaries, Fallopian tubes, uterus, cervix, and vagina. Ovaries are responsible for the production of the ovum and secrete both estrogen and progesterone. When an ovum is developing in an ovary, it is encapsulated in a sac known as an ovarian follicle. On maturity of the ovum, the follicle and the ovary’s wall rupture, allowing the ovum to escape and enter the fallopian tube to reach uterus. The uterus is a muscular organ useful to accept a fertilized ovum, which becomes implanted into the endometrium. The cervix is the neck of the uterus, which protrudes through the upper anterior vaginal wall. The vagina is a fibromuscular canal that connects the upper part of female genital tract to the outside of the body and represents the portal of entry of pathogenic microorganisms. The epithelial mucosa of the lower genital tract is extensively colonized by commensal microorganisms, while the tissues of the upper genital tract are generally considered to be sterile (1). However, bacterial colonization of the upper genital tract of healthy asymptomatic women remains a somehow controversial issue (1).

The vaginal tract is colonized by microorganisms, recognized as the vaginal microbiota (VM). These microorganisms, in addition to a complex synergism among secretion’s proteins and peptides, epithelial, and immune cells, perform a pivotal role in the defense of female genital tract against infectious and inflammatory processes.

In the state of mucosal health, the various components are in balance. The rupture of mucosal homeostasis determined by the alteration of one of the various actors often results in an increased host susceptibility to infections. Healthy VM is dominated by *Lactobacillus* spp., but other microorganisms can be present at lesser extent (*Gardnerella, Prevotella, Streptococcus, Ureaplasma, Peptostreptococcus, Staphylococcus, Corynebacterium, Clostridium, Mycoplasma, Enterococcus, Bacteroides, Escherichia, Bifidobacterium, Veillonella*, and *Candida*) (2–6).

Over 20 species of *Lactobacillus* have been detected in the vagina. However, in the majority of women, the healthy vaginal microflora contains one or two *Lactobacillus* species among *Lactobacillus crispatus, Lactobacillus gasseri, Lactobacillus jensenii*, and *Lactobacillus iners* (7, 8). Currently, the role of *L. iners* in vaginal health is still unclear (9). Indeed, *L. iners* has been recently detected in both dysbiotic and healthy women, and its presence and amount are inversely correlated with *L. crispatus* (8, 10, 11).

Lactobacilli are involved in maintaining the healthy vaginal environment by counteracting overgrowth of other resident microorganisms (12). Lactobacilli can also colonize the human cervix. In different studies, a range of 29–52% of women resulted colonized by lactobacilli in the cervix, and within a single subject, usually the same *Lactobacillus* strains colonized both the cervix and the vaginal tract (13–15). Lactobacilli exert their protective effects by several mechanisms: (i) microbial competition for the nutrients and for adherence to the vaginal epithelium; (ii) reduction of the vaginal pH by the production of organic acids, especially lactic acid, through the degradation of glycogen released by vaginal cells thus exerting selective antimicrobial activity against non-resident microbiota; (iii) production of antimicrobial substances, such as bacteriocins and hydrogen peroxide (H_2_O_2_) able to suppress the growth of several microorganisms; and (iv) modulation of the local immune system (16). Homeostasis of vaginal environment results from complex interactions and synergies among the host and different microorganisms that colonize the vaginal mucosa, and the maintenance of high numbers of resident lactobacilli is an effective hallmark of woman’s health and a well-organized protection against pathogens causing sexually tranmitted infections (STIs). Abnormal VM involving a strong reduction or disappearance of lactobacilli characterizes a pathologic condition known as bacterial vaginosis (BV) that afflicts fertile, premenopausal, and pregnant women with an incidence rate ranging from 20 to 50% (17). BV is a polymicrobial clinical syndrome resulting from the replacement of the normal *Lactobacillus* spp. with high number of anaerobic bacteria such as *Gardnerella vaginalis, Prevotella* spp., *Mobiluncus* spp., *Ureaplasma, Mycoplasma*, and other fastidious or not culturable anaerobes (5, 18). In BV, the overgrowing anaerobes produce compounds such as polyamines and other molecules capable of inducing the release of pro-inflammatory cytokines such as IL-1β, IL-6, and IL-8 (19, 20). BV represents an independent risk factor for severe reproductive tract sequelae associated with pelvic inflammatory disease and tubal factor infertility (21, 22). Changes in the VM have been also associated with obstetrical complications such as late miscarriage and premature birth (23), thus exerting a profound impact also on the health of newborns. Moreover, women with *Lactobacillus* poor flora show an increased susceptibility to sexually transmitted pathogens. Several studies indicate that abnormal VM lacking lactobacilli is associated with the acquisition of infections by *Neisseria gonorrhoeae, Chlamydia trachomatis*, and *Trichomonas vaginalis* (24–28).

Furthermore, the alterations in VM are associated with increased risk of acquiring viral sexually transmitted diseases (STDs). Indeed, longitudinal and cross-sectional studies demonstrated the association between altered VM and the increased prevalence/incidence of many viral STIs such as human immunodeficiency virus (HIV), herpes simplex virus (HSV), human papillomavirus, and cytomegalovirus infection (29).

In addition to lactobacilli, the cervicovaginal fluid (CVF) exerts a significant microbicidal activity against Gram-positive and Gram-negative bacteria, fungi, and certain viruses as well as an anti-inflammatory activity through several peptides and proteins, all characterized by common cationic features (30). The main antimicrobial peptides and proteins present in the CVF are shown in Table 1 (31–46). These peptides and proteins act through different mechanisms: (i) microbial lysis; (ii) depletion of environmental nutrients essential for microbial growth; (iii) competitive binding to host cells; (iv) degradation of negatively charged microbial surface components; (v) interference with host cell signaling pathways; and (vi) modulation of inflammation and other functions involved in host defense (34, 47). The bactericidal activity of many of these compounds is strictly associated with their cationic features. The concentration of some of these molecules in CVF is lower than that required for *in vitro* inhibition of pathogens; however, it is known that several antimicrobials display synergistic effects. Indeed, human β defensin 2 and cathelicidin antimicrobial peptide LL-37 (48), secretory leukocyte protease inhibitor (SLPI) and lysozyme (49), and lactoferrin (Lf) and lysozyme (49) display synergistic effects that potentially increase innate immune protection in the female reproductive tract (50).

**Table 1:** Antimicrobial peptides/proteins of the female genital tract.

Peptide/protein	Source	Antimicrobial effect	Reference
SLPI	Epithelial cells	Gram-positive and Gram-negative bacteria	(31, 32)

α-defensins	Neutrophils	Gram-positive and Gram-negative bacteria	(31, 33–35)
	Macrophages	Enveloped and non-enveloped viruses	
	Monocytes		
	Epithelial cells		

β-defensins	Epithelial cells	Gram-positive and Gram-negative bacteria	(34, 36)
	Neutrophils	Fungi	
	Macrophages	Enveloped and non-enveloped viruses	
	Monocytes		
	Dendritic cells		

Trappin2-Elafin	Epithelial cells	Gram-positive and Gram-negative bacteria	(37, 38)
	Neutrophils	Fungi	
		HIV	
		HSV-2	

Calprotectin	Epithelial cells	Gram-positive and Gram-negative bacteria	(39, 40)
	Neutrophils		
	Monocytes		

Cathelicidin	Neutrophils	Gram-positive and Gram-negative bacteria	(41)
	Epithelial cells

Lysozyme	Macrophages	Gram-positive bacteria	(42, 43)
	Monocytes	HIV	
	Neutrophils	HSV	

Lactoferrin	Epithelial cells	Gram-positive and Gram-negative bacteria	(44–46)
	Neutrophils	Enveloped and non-enveloped viruses	

*HIV, human immunodeficiency virus; HSV, herpes simplex virus; SLPI, secretory leukocyte protease inhibitor*.

Many of these antimicrobial/immunomodulatory compounds appear to be under hormonal control (31). In CVF α and β defensins, SLPI and lysozyme levels are high during the proliferative phase, greatly decrease at mid-cycle/ovulation, and increase again during the late secretory phase.

Lactoferrin, belonging to transferrin family, is a multifunctional glycoprotein of about 690 amino acids and a MW of 80 kDa. Lf chelates two Fe (III) per molecule with high affinity (Kd ~ 10^–20^ M) until very low pH as 3.0, characteristic of infection sites (44, 51, 52). Lf, abundantly found in most biological fluids of mammals, is synthesized by exocrine glands, many mucosal epithelial cells and released by neutrophils during inflammation. The highest level of the human Lf (hLf) is found in colostrum (7 mg/ml), while decreases in mature milk (1.5–4.0 mg/ml). In the tears, hLf is detected at low concentration (about 2.0 mg/ml), while in saliva, small intestine, earwax, vaginal fluid, amniotic fluid, upper airway fluid, seminal plasma, and the cervical mucus at very low levels (<0.1 mg/ml) (53). In particular, the concentration of hLf in human vaginal fluid corresponds to 1–3 µg/ml, while it is extremely high (100 µg/ml) in the cervical mucus plug (30, 54, 55). Of note, a total number of 10^6^ neutrophils release 15 µg of hLf in sites of inflammation and infection (53).

In human body fluids, the concentration of free available iron must not overcome 10^–18^ M to avoid microbial multiplication and to hinder the precipitation of insoluble ferric hydroxides as well as the formation of reactive oxygen species. HLf, by its iron-binding ability, guarantees that free available iron does not exceed 10^–18^ M. HLf and bovine milk derivative Lf (bLf) possess high homology of sequence. From three-dimensional structure, Lf is folded into two homologous lobes, each structured in two domains (N1 and N2, C1 and C2). One Fe (III) ion is chelated by each lobe. When Lf is completely iron saturated, its conformation appears in a closed state, more resistant to proteolytic enzymes than the unsaturated open form (51). The low iron availability (10^–18^ M), hindering microbial growth, is a signal of health and wellness, while iron concentration higher than 10^–18^ M favors not only microbial replication but also the biofilm formation (56, 57) and persistence (44).

Interestingly, Wiesner and Vilcinskas have reported that proteins and peptides of mucosal secretions possess several functions (58). Accordingly, hLf and bLf are multifunctional glycoproteins effective against bacteria, mycetes, viruses, and parasites, possessing also anti-inflammatory and immunomodulatory properties (44). In particular, bLf, available in large quantities and recognized by Food and Drug Administration (FDA, USA) as a safe substance, is the main Lf used in *in vitro* studies (44) as well as in clinical trials (52, 59–62) and in mice (63).

The level of vaginal hLf and others antimicrobial peptides change in response to microbial infections. It has been demonstrated that hLf and defensin levels increase in genital secretion of women with *C. trachomatis, N. gonorrhoeae, T. vaginalis*, or *Candida* spp. infection and BV in comparison to healthy condition (64, 65).

## Antimicrobial Activity of Lactobacilli and Lf

### Lactobacilli

The innate defense system of the female mucosal genital tract involves a complex interaction among the healthy vaginal flora, immune cells, and several proteins that defend the host from pathogens. It is broadly suggested that the crucial role of vaginal lactobacilli is to protect female genital tract through the production of lactic acid responsible for low vaginal pH that inhibits sexually transmitted pathogens. Lactic acid is in equilibrium with lactate anion. The former is the predominant form in healthy vaginal conditions and low pH (<4.5), thus exerting antimicrobial activity against pathogens. Lactate anion predominates at higher pH (>4.5) conditions in women with dysbiosis (66). *In vitro* experiments demonstrated that at physiological concentrations (55–111 mM) of lactic acid and at pH 4.5, BV-associated bacteria such as *G. vaginalis* and *Atopobium vaginae* were inactivated without effects on typical vaginal species of lactobacilli (67). Furthermore, inactivation of BV-associated species was dependent on lactic acid itself rather than pH, since a pH value of 4.5 determined by other acids was significantly less microbicidal. Recent *in vitro* studies, however, demonstrated that *C. trachomatis* multiplication is inhibited by different strains of vaginal lactobacilli, independently from pH alterations (68). *C. trachomatis*, an obligate intracellular pathogen, responsible for the most common bacterial STD worldwide, causes acute and chronic infections. Unlike acute infections, which can be cured with oral or topical administration of antibiotics, chronic infections are difficult to eradicate and need prolonged therapies, thus increasing the risk of developing antibiotic resistance (69). Therefore, novel alternative therapies are needed. The difficulty in finding new agents against *C. trachomatis* infection resides in the complex life cycle of this peculiar pathogen. In fact, *C. trachomatis* has a unique biphasic developmental cycle, alternating between the extracellular infectious elementary bodies (EBs), metabolically inactive, and the intracellular non-infectious reticular bodies (RBs), which are metabolically active. It has been recently demonstrated that vaginal lactobacilli inhibit EBs adhesion to epithelial cells as well as the intracellular RBs replication (68). The effect on the early phases of infection was related both to co-aggregation between lactobacilli and *C. trachomatis* and to competition for epithelial cell adhesion. The inhibition of chlamydial infection by lactobacilli was strain and dose dependent, suggesting that the strains and the amount of lactobacilli in the vagina are responsible for the protection from chlamydial infection.

Lactobacilli have been demonstrated to protect lower female genital tract also from *N. gonorrhoeae* infection, the second most common bacterial STI. The interaction between bacteria and host cells determines the success of the pathogen mucosal colonization or its elimination through the continuous fluid flow. In this respect, Vielfort et al. (15) showed that lactobacilli compete with *N. gonorrhoeae* for adhesion to human cervical cells. It has been also demonstrated that *L. jensenii* ATCC 25258 could reduce both adhesion and invasion of *N. gonorrhoeae*, whereas *L. gasseri* ATCC 33323 could displace adherent gonococci from the cell surface (70).

The protection exerted by healthy VM toward viral infections can be ascribed to a direct virucidal effect or to the maintenance of natural defense factors present in the vaginal milieu. Some mechanisms have been suggested by results obtained from both *in vitro* experiments and clinical observations in infected women (29). *Lactobacillus* metabolites possessing antimicrobial activity may be directly protective against viral infections.

Hydrogen peroxide (H_2_O_2_) produced by lactobacilli plays an important role as a natural microbicide within the vaginal ecosystem due to its toxic activity against a number of microorganims and viruses, including HIV-1 (71) and HSV-2 (72). It has been observed that a range of 70–95% of lactobacilli present in the vaginal flora of healthy women produce H_2_O_2_. This percentage drops to 5% in women affected by vaginal infections (73).

The physiological acid vaginal pH value (≤4.5) determined by lactobacilli inactivates HIV (74) and HSV-2 (75). In addition, HSV-2 is inactivated by lactic acid concentrations leading to pH values similar to the ones detected in the healthy human vagina (72). Several compounds released from lactobacilli can impair the efficiency of target cells in supporting viral replication. A non-protein cell wall component extracted from a vaginal strain of *Lactobacillus brevis* strongly reduces HSV-2 replication in cell culture (76), whereas acid *Lactobacillus* metabolic products decrease activation of T lymphocytes, with a consequent lower lymphocyte susceptibility to HIV-1 infection (77).

A healthy VM contributes to the maintenance of the natural defense mechanisms from invading pathogens. The gel layer coat of the vaginal and cervical mucosa represents a physical barrier that hinders viral binding to cell membrane receptors, thus protecting women from viral infections. Indeed, *in vitro* studies demonstrated that HSV could be trapped into the viscous cervical mucus (78). BV-related microorganisms are able to produce higher levels of mucin-degrading enzymes, such as mucinase and sialidase, in comparison to lactobacilli-dominated healthy vaginal flora (79–81). Therefore, an increased degradation of the protective mucus layer may promote binding of HSV-2 and other viruses to the underlying epithelial cell receptors. Further studies have demonstrated that vaginal lactobacilli are able to inhibit the first steps of HSV-2 infection in cell culture (72, 76). The antiviral activity exerted by the presence of lactobacilli during HSV-2 binding to the cell membrane is strain dependent and appears directly related to the adhesion capacity of *Lactobacillus* strains (82).

In conclusion, several mechanisms may be involved in the antimicrobial effect of vaginal lactobacilli: interference with microorganisms in the process of adhesion or entry into host cells, production of metabolites with a direct antimicrobial effect, production of compounds able to inhibit obligate intracellular pathogen replication, and contribution to the maintenance of natural defense factors present in the vaginal milieu.

### Lactoferrin

As already discussed, hLf is one of the most important defense proteins of CVF. In fact, endogenous hLf has been found increased in the genital fluid of women affected by *Neisseria gonorrheae, C. trachomatis*, and *T. vaginalis* infections and/or vaginal dysbiosis (64). In this respect, hLf released by neutrophils recruited *in situ* could represent a marker of non-healthy conditions and one of the mediators involved in counteracting the inflammatory mileu.

The first function of hLf, recognized *in vitro*, was the bacteriostatic activity depending on its ability to sequester iron necessary for bacterial survival and growth (83). HLf and bLf establish a battle for iron acquisition with pathogens, capable to counteract these iron-binding proteins by synthesizing siderophores, small high affinity iron-chelating molecules, or through iron acquisition from other sources (65). As a matter of fact, *G. vaginalis*, lacking of siderophores, acquires iron by the lysis of erythrocytes, using hemoglobin as iron source. This is consistent with the observation that *G. vaginalis* level increases during menses (6).

Moreover, independently from iron-binding ability, bLf exerts several antibacterial activities: (i) bacterial lysis through its binding to lipopolysaccharide (LPS); (ii) inhibition of bacterial adhesion to the epithelial cells; and (iii) inhibition of the entry into host cells by facultative or obligate intracellular bacteria through competitive binding to host cells and/or to microbial surface components [(44) and references therein (46, 84)]. Of note, facultative intracellular pathogens require intracellular nutrients, including iron, for replication in mammalian cells, and obligate intracellular *C. trachomatis* is no exception (85).

A preparation of bLf, iron saturated at 20% to consent further iron chelation, was utilized in *in vitro* model to check its antichlamydial activity (84). Similar to that observed using vaginal lactobacilli, the incubation of cell monolayers with bLf before the infection or at the moment of the infection significantly inhibited the adhesion and entry of *C. trachomatis* into epithelial cells. Therefore, the inhibition of *C. trachomatis* infectivity by bLf was dependent on its interaction with the cell surface and especially with glycosaminoglycans and heparan sulfate proteoglycans (86), which are potential receptors for *C. trachomatis* adhesion (87). Conversely, the preincubation of bLf with *C. trachomatis* EBs did not influence its infectivity, supporting the idea that the specific interaction between bLf and epithelial host cells could be the sole mechanism responsible for the inhibition of *C. trachomatis* invasion (84). Recently, the inhibition of IL-6 and IL-8 synthesis by bLf has been demonstrated in *in vitro* model, mimicking the *in vivo* chlamydial infection. BLf, added to infected cells 3 h postinfection, produced a significant decrease of IL-6 and IL-8 without any effect on the number of intracellular *Chlamydia*. Similarly, IL-6 levels were reduced when bLf was added 3 h postinfection to epithelial monolayers infected with other facultative intracellular bacteria or to LPS-stimulated macrophages (88–90). The anti-chlamydial activity of bLf related to its anti-inflammatory function has been shown *in vivo*. In a pilot study, 7 of 176 pregnant women showing cervical specimens positive for *C. trachomatis* were treated with the intravaginal administration of bLf (100 mg) every 8 h for 30 days. Interestingly, after 1 month, six women resulted negative for *C. trachomatis* and showed significant decreased IL-6 levels in their CVF (84). Similar to what observed in *in vitro* model, intravaginal administration of bLf seems to act by protecting mucosal host cells against the adhesion and entry of chlamydial EBs, which are released extracellularly after redifferentiation of RBs to EBs. The decrease of IL-6 levels could be a marker for the inhibition of *C. trachomatis* EBs infection of host cells due to the presence of bLf. In other words, bLf protects host cells and prevents the early phase of infection by EBs. Unlike lactobacilli (68), bLf does not affect the replication of RBs (84). The potential influence of exogenous bLf on microbial communities populating vagina has been recently investigated. Vaginal bLf administration to 60 women with BV has been shown to be able to modify VM composition. In fact, the treatment induced a reduction of BV-associated *Gardnerella, Prevotella*, and *Lachnospira* genera as well as an increase of *Lactobacillus* species (91). These data suggest the therapeutic potential of bLf in counteracting female genital tract diseases. Indeed, it would be relevant to unveil the molecular mechanisms as well as the immunological changes accompanying bLf effects on microbiota.

Furthermore, bLf antiviral activity, verified against both enveloped and naked viruses, is exerted in the early stage of infection, thus inhibiting viral binding and entry into the host cell. This activity is mainly due to bLf binding to heparan sulfate glycosaminoglycan cell receptors or viral particles or both (45). Similar to viral particles, the inhibition of *Plasmodium* endocytosis is attributed to the interaction between bLf and both cell surface heparan sulfate and lipoprotein receptor-related protein (92–95).

In this respect, bLf represents the most relevant protein symbolizing a brick in the wall of natural non-immune defenses of human mucosal fluids against microbial infections (44).

## Lactobacilli and Lf Host Immune System Modulation

### Lactobacilli

It is well known that lactobacilli are endowed with health-promoting and immunomodulatory properties. Along with bifidobacteria, they have been proposed as candidates for prevention and/or treatment of allergy, colitis, infections, and other inflammatory conditions (96). Some *Lactobacillus* strains have been also proposed as vaginal microbicide candidates against STI (e.g., *N. gonorrhoeae, Candida albicans*, and HIV). Besides mechanisms related to the bacterium itself (e.g., enhancement of epithelial barrier function and competition with pathogens), the capability to redirect the immune response underlies many of the beneficial effects of lactobacilli. *In vitro* data demonstrate that *L. crispatus* (97–99) and *L. jensenii* (100) act not only through colonization of epithelial cells but also influencing the cytokine secretion pattern. In particular, upon recognition through Toll-like receptor (TLR) 2/6 and 2/4, NF-κB signaling is activated without induction of pro-inflammatory mediators (IL-1β, IL-1α, and TNF-α). Furthermore, secretion of cytokines as IL-8 is inhibited, while production of IL-10, IL-6, and defensins can be induced ensuring homeostasis of immune responses. Although innate immunity is the first level to be influenced by the probiotic interaction with mucosal epithelium, other cells (e.g., dendritic cells) can be shaped by lactobacilli to skew adaptive responses. An example is represented by *L. crispatus* SJ-3C-US strain, which was shown to confer anti-inflammatory properties to dendritic cells by inducing upregulation of IL-10 production and induction of regulatory T cells (101). As mentioned above, a key metabolite produced by lactobacilli is lactic acid. Besides its antimicrobial properties, many of the immune modulation mechanisms exerted by lactobacilli can be ascribed to this compound. In particular, lactic acid has been shown to induce an anti-inflammatory response from vaginal and cervical epithelial cells by inhibiting IL-6, IL-8, RANTES, and TNF-α secretion stimulated by TLR agonists used to mimic pathogen-associated molecular patterns (PAMPs) from microbes (102). Given that IL-6, IL-8, and TNF-α are known to promote replication of HIV through activation of NF-κB transcription in HIV target cells, lactic acid produced by lactobacilli in the vaginal environment could be relevant in the context of viral infection acquisition. Furthermore, the anti-inflammatory cytokine IL-1RA was induced by lactic acid treatment of cervicovaginal epithelial cells. All these anti-inflammatory effects were mediated by both l- and d-lactic acid isomers and by the protonated form which predominates in healthy conditions with low values of vaginal pH (102, 103). Very few data about the immunological changes associated with benefits induced by lactobacilli administration are available in the vaginal tract *in vivo* in both physiological and pathological conditions. *Lactobacillus salivarius* CRL 1328 and *L. gasseri* CRL 1263 have been proposed as good candidates to keep a balanced microbiota and immune surveillance. Indeed, in a murine model set up to evaluate the benefits of lactobacilli and their effects on the mouse vaginal mucosa and innate immune cells, lactobacilli inoculation did not modify the amounts of granulocytes and macrophages in vaginal washings (104). In humans, it has been shown that administration of probiotic *Lactobacillus* vaginal tablets produces a significant reduction in the levels of vaginal IL-1β and IL-6 cytokines demonstrating the capacity of lactobacilli to modulate the production of inflammatory cytokines in both women with BV and women with healthy vaginal flora (105, 106).

A link between oral probiotic administration and VM/immune markers has been recently demonstrated by Vitali et al. on pregnant women (107). The authors investigated the effects of dietary supplementation with VSL#3 probiotic mixture containing eight species of *Lactobacillus, Bifidobacterium*, and *Streptococcus* on the VM during late pregnancy. Interestingly, no changes in the bacterial counts of the most represented populations were revealed upon probiotic administration. However, the probiotic mixture was able to change the composition of less abundant vaginal microorganisms by avoiding the reduction of *Bifidobacterium* and the increase of *Atopobium* recorded in the last trimester of pregnancy in control healthy women. Significant modifications of the local immune system were also associated with the consumption of the probiotic showing anti-inflammatory effects. In particular, vaginal levels of IL-4 and IL-10 were maintained in balance compared to the reduction observed in control group. Furthermore, vaginal levels of eotaxin, a pro-inflammatory chemokine, were reduced upon probiotic dietary supplementation.

### Lactoferrin

Similar to lactobacilli, also bLf exhibits effects on the host immune system, ranging from inhibition of inflammation to promotion of both innate and adaptive immune responses (108). Innate immunity is shaped by endogenous hLf through its interaction with PAMPs and/or pattern recognition receptors (PRRs) expressed by host cells. *In vitro* studies demonstrated that carbohydrate chains of hLf make it able to interact directly with TLR4 resulting in moderate activation of TLR4 associated pathways (109). LPS is a typical PAMP, which is bound by hLf, resulting in the inhibition of cell activation and inflammatory responses (110, 111). It has been proposed that *in vivo* hLf inhibits LPS-stimulated TLR4 signaling and depresses endotoxemia (112). In particular, upon LPS binding, hLf acts in reducing TNF-α, IL-1, and IL-6 production by immune cells (macrophages, neutrophils, and lymphocytes), as well as IL-8 release by endothelial cells (113). It has been demonstrated that levels of hLf are increased in inflammatory diseases such as rheumatoid arthritis (114), severe acute respiratory syndrome (115), inflammatory bowel disease (116), and as mentioned above, some STD and BV (64). These observations suggest that hLf could be used as a clinical marker of inflammatory conditions. Besides PRRs and PAMPs binding, hLf displaces proteases from heparin in mast cells thus playing antiallergic effects (117). Furthermore, hLf competes with IL-8 through the binding to proteoglycans on endothelium, thus interfering with neutrophils recruitment to the site of inflammation (118). HLf has been shown to bind also DNA in the neutrophil extracellular traps (NETs), and this capability plays a key role in the context of NETosis, which is the NETs production by neutrophils. HLf adheres to the DNA structures released due to chromatin decondensation and spreading exerting its antimicrobial properties (119). *In vitro* experiments showed that recombinant hLf is able to induce maturation of antigen-presenting cells such as dendritic cells, thus suggesting that it can represent a link in shaping adaptive immunity (120). Depending on the external stimulus (pathogens, allergen, tumor antigens, etc.) and host immune status, hLf can modulate IL-4, IL-2, IL-12, or IFN-γ levels, thus providing different outcomes: strong Th1 polarization (infections, tumor), reduction of excessive Th1 responses, and correction of Th1/Th2 balance (allergy, autoimmunity). Furthermore, Lf is able to support proliferation of T cell precursors and their differentiation. Besides its role in cellular-mediated immunity, hLf influences activation of B cells, thus playing a role also in humoral responses (113).

The effects of exogenous Lf on immune responses have been evaluated in different *in vitro* systems. Different epithelial cell monolayers infected with various facultative or obligate intracellular pathogens produce pro-inflammatory cytokines and the addition of bLf significantly decreased IL-1β, IL-6, IL-8, and NF-kB levels (84, 88, 121, 122). The results obtained in different *in vitro* models and in various clinical trials confirm the bLf ability in downregulating pro-inflammatory cytokine synthesis. It has been demonstrated that exogenous bLf localizes into cell nucleus, thus acting as transcriptional factor and inhibiting pro-inflammatory cytokines (52, 122–124). Although the mechanisms by which bLf exerts its anti-inflammatory activity are still under debate, recently, the bLf ability to decrease the high levels of IL-6 in CVF seems strictly related to its capacity to restore iron homeostasis disorders (52, 84). Therefore, Lf is not only a key element in the host defense system (44, 125, 126) but also a pivotal component that is able to regulate the inflammatory response and iron homeostasis (52, 60–62). Recently, bLf is emerging as an attractive molecule for treating inflammation by ranging pro-inflammatory macrophagic phenotypes M1 to regulatory/anti-inflammatory M2 phenotypes (90).

## Women Life: Estrogens, Lactobacilli, and Lf

### Lactobacilli

Defenses of female mucosal genital tract are largely under the influence of hormones and paracrine production of various cytokines. Vaginal environment undergoes overtime shifts in the representation and abundance of microbial key species that are influenced by factors that may include age, hormonal fluctuations, sexual activity, use of medication, and hygiene (12). The vagina is lined by stratified not keratinizing squamous epithelium, which is variable in thickness and structure depending on life stages. The vaginal epithelium consists of three cell layers: superficial, intermediate, and basal capable of storing glycogen under the influence of estrogen. In the pre-pubertal and the postmenopausal women, the epithelium is thin and characterized by a basal layer of cells and several layers of parabasal cells. This thin atrophic epithelium is susceptible to infection and frequently shows degenerative and inflammatory changes. Vaginal epithelium reflects the hormonal changes of the menstrual cycle with increased mitosis of the basal layers. Under the influence of estrogen in the proliferative phase of the cycle, the whole epithelium thickens and is multilayered. During the secretory phase of the cycle, the intermediate layers become thick and the cells stuffed with glycogen. Therefore, the glycogen content of the vaginal epithelium co-variates with estrogen levels. Breakdown of glycogen by resident healthy VM produces an acid pH in the vagina, which deters infections. Indeed, reproductive-age women carry *Lactobacillus* species (predominant lactic acid bacteria) and genera of *Streptococcus* and *Atopobium*, which conserved the ecological function of lactate production in the vaginal microbiome (127).

It is well demonstrated that fluctuations in the VM occur not only based on intercourse and infections but also during menstrual cycle. In general, high levels of estradiol may favor a lactobacilli-dominant environment, especially *L. crispatus, L. gasseri*, and/or *L. jensenii* (128), which can be underrepresented in low estrogen conditions such as the beginning of a menstrual cycle or in postmenopausal women. In one study, *L. crispatus* appears to decline during menses, while *G. vaginalis* increases along with *L. iners* and subsequently the concentration of both species decreases after menses (6). In other studies, *L. crispatus* has also been reported to decline 100-fold during menses, while the numbers of *L. iners* strongly increase (10, 11, 129).

Recently, cultivation-independent methods have highlighted the complexity and temporal variability of the VM (8, 130). In particular, Gajer et al. described temporal changes in the composition of VM in reproductive-age women within a 16-week period (129). In general, the highest variability of microbiota community was associated with menses. For example, a subject can show a community dominated by *L. crispatus*, which could be replaced by *L. iners* during menses or by *Streptococcus* spp. in a different subject. The same community could then revert to a community dominated by *L. crispatus* at the end of menses. Moreover, the results also showed how some bacterial communities changed greatly over short time periods, while others were more stable. Hormone levels were combined with diversity data showing that an increase in estradiol and progesterone corresponded to decreased microbial variability. Vaginal metabolome data added information about community function, which was maintained despite changes in its composition. Indeed, shifts in community composition involved only changes in the relative dominance of a little number of different bacteria that are able to produce lactic acid (129).

Interestingly, a recent study performed to investigate timing and sequence of changes that occur in the vaginal and vulvar microbiota during puberty showed that VM of perimenarcheal girls resembles those of reproductive-age women (131). In fact, *L. crispatus, L. iners, L. gasseri, L. jensenii*, and, in some subjects, *Streptococcus* spp. were dominant in the microbiota of girls before the onset of menarche in the early to middle stages of puberty. Further studies should be performed to increase knowledge about the link between estrogen, vaginal glycogen levels, lactic acid bacteria abundance, and vaginal pH.

Other important fluctuations in the vaginal microbiome are recorded during pregnancy. Aagaard et al. showed that microbiome was enriched in *L. iners, L. crispatus, L. jensenii, and L. johnsonii* (132). The increase in lactobacilli may be due to the increase in estrogen levels that occurs during pregnancy although further investigations are needed to better understand the relationship between specific species of *Lactobacillus* and estrogen levels. However, another study shows that *L. crispatus* and *L. iners* dominate the vaginal flora as pregnancy progresses and maternal age seems to be important for the dominance of *L. crispatus* or *L. iners*, with *L. iners* being dominant in older gravidae (133).

As mentioned above, menopause usually represents a phase in which lactobacilli levels are low, but it seems that their implication is even more pronounced involving other features. Actually, inverse correlation has been found between *Lactobacillus* levels and vaginal dryness, a common condition of postmenopausal period, which was shown to be associated with changes in vaginal epithelial cell integrity and inflammation (134).

Besides the endogenous hormonal fluctuations, clinical evidences demonstrate that the use of hormonal contraceptives is also able to induce changes in VM, thus influencing the susceptibility to STI and BV. Therefore, STI and BV (which in turn predisposes to STI) depend, in part, on modification of vaginal bacterial communities induced by some contraception methods. Clinical trials based on Nugent score endpoint and questionnaires have revealed a reduced BV rate in women who use estrogen-containing contraceptives (135, 136). The effects of progestin-containing contraceptives such as depot medroxyprogesterone acetate (DMPA) and levonorgestrel are less clear. In a systematic review of 36 eligible studies, authors have shown that combined oral contraceptives (COCs; combination of an estrogen and a progestin) and DMPA reduce BV by a range of 10–20 and 18–30%, respectively (137). Accordingly, in a recent retrospective study on 682 fertile women, based on 16S rRNA sequencing, authors found that women using COC or DMPA showed a reduced colonization by BV-associated bacteria compared to women using condoms. In the same study, women using progestin-based therapies have a significantly higher abundance of taxa associated with a dysbiotic VM. On the other hand, COC users have a lactobacilli-dominated VM and a higher proportion of H_2_O_2_-producing *Lactobacillus* species (*L. crispatus, L. gasseri*, and *L. jensenii*) correlating with vaginal health compared to progestin-containing contraceptives (138) Differences in the results reported in other studies, which do not reveal any changes in vaginal bacterial communities associated with progestin use, could be due to the different study population characteristics (e.g., ethnicity of subjects) and/or the design of the study itself such as the specific BV status examination (139, 140). In women with BV, VM is not the only level that hormonal contraception acts on. In fact, changes in genital tract immunity by effect of hormonal contraceptives have been shown in terms of both suppression and activation of responses. In a study on 81 women, cervical secretions contained lower levels of pro-inflammatory molecules (TNF, IFN-γ, and GM-CSF) in subjects with BV using hormonal contraceptives compared to those not using them (141). On the other hand, an increase of inflammatory cytokines (MIP-1α, MIP-1β, IL-6, IL-8, IP-10, and RANTES) has been associated with hormonal contraception in a different cross-sectional analysis including 376 African women (142). Conflicting data existing in these studies on immunomodulatory potential of hormonal contraception in female genital tract are complicated by their variability in terms of *in vitro* versus *in vivo* models used and sample tested (plasma or blood *versus* cervical fluid). However, the investigation on these features may represent a key point in understanding the association between hormonal contraception and HIV acquisition. In fact, women using progestin DMPA, but not those who use COC have been found to be at significantly increased risk of HIV infection compared to women not using hormonal contraception (143). In a more recent case–control selection of specimens from a large, prospective, clinical study, the same authors have investigated on innate immunity mediators in cervical samples collected from 199 women at their visit before HIV seroconversion and matched visits from 633 women remaining HIV uninfected. Higher levels of pro-inflammatory markers such as RANTES have been found in HIV seroconversion and in DMPA users, suggesting a possible role of this cytokine in the association between the contraceptive and HIV risk acquisition (144). The possible explanation could be that the upregulation of RANTES, observed in women using DMPA and women with BV, may simultaneously block the CCR5 cellular HIV receptor but also facilitate transmission through recruiment of target cells. Also other STI can be influenced by hormonal contraception, such as candidiasis, which has been found increased in women using COC but not DMPA (137). Taken together, these observations suggest that altered immune responses by effect of hormonal contraception may predispose to infections from pathogens. In cases of a pre-existing condition of dysbiosis or specific cervicovaginal infection, suppression or activation of immunity by pathogens could increase the risk of infections by cumulative action with exogenous hormones.

### Lactoferrin

Similar to the estrogen-induced changes in the species and number of lactobacilli, the hLf concentration fluctuates accordingly to circulating estrogen levels (145–147). In addition to the hLf levels produced by uterine epithelium and released in CVF, the synthesis of IgA and IgG is also modulated by estrogen and progesterone, thus exerting an immune protection against sexually transmitted pathogens (148).

**Figure 1:**
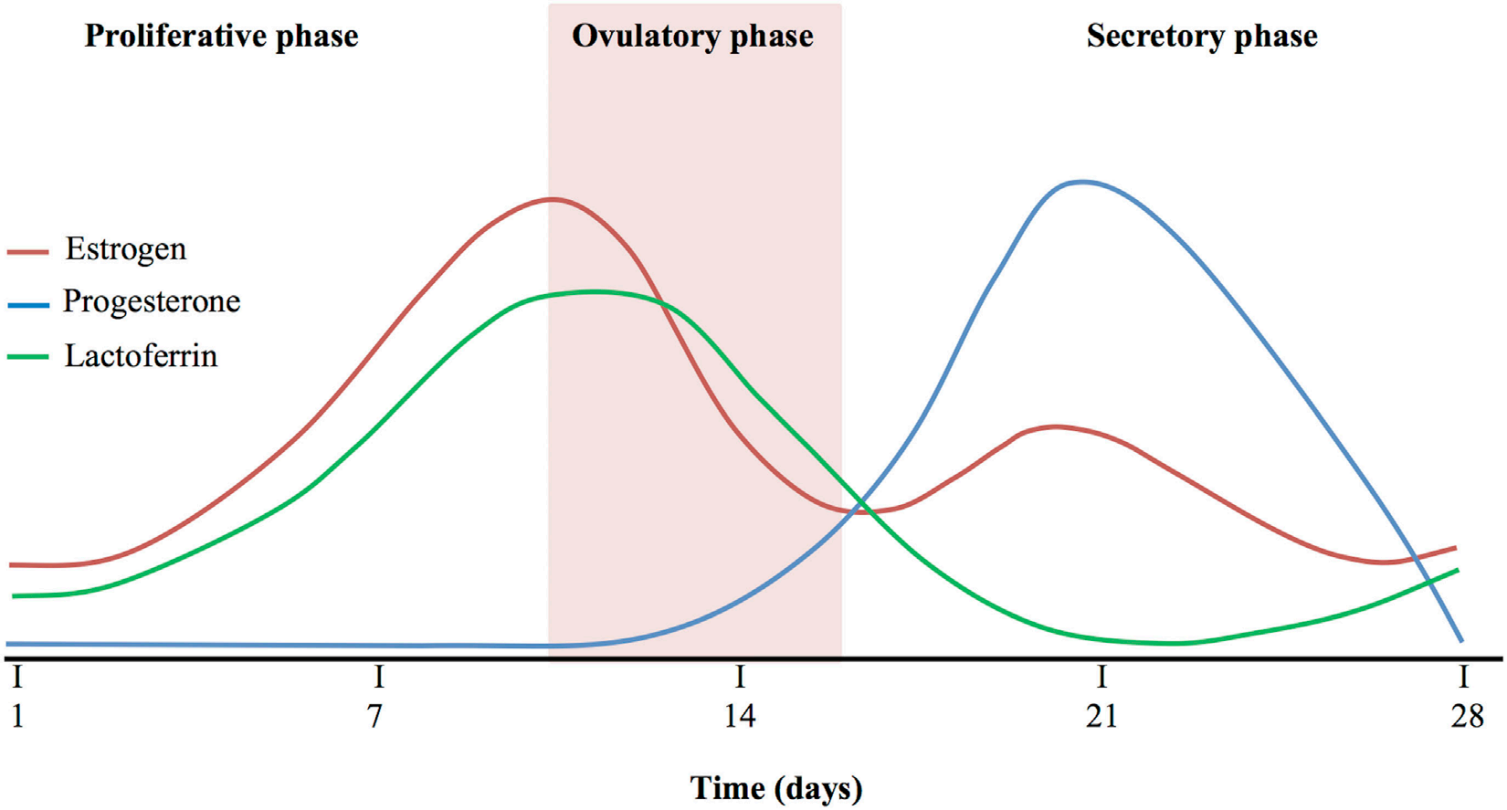
Comparison among estrogen, progesterone, and Lactoferrin levels.

Figure 1 shows a comparison among estrogen, progesterone, and Lf levels during the proliferative, ovulatory, and secretory phase. The hLf levels increase in line with estrogen production, reaching the highest levels during ovulatory phase, while they are inversely correlated with progesterone levels. As a matter of fact, secretory phase accordingly to the increase of progesterone shows the lowest levels of hLf. At the end of the secretory phase, hLf returns to increase in parallel to the progesterone decrease (145–149).

Obviously, the use of oral contraceptives by decreasing estrogen synthesis suppresses the production of hLf as well as immunoglobulins for the duration of hormone exposure, thus possibly increasing the susceptibility of women to infections (148).

In rats, pretreatment with progesterone prior to exposure to *Chlamydia thracomatis* infection induced a persistent infection (150). Furthermore, the rats treated with progesterone were also found to be more vulnerable to chlamydial intrauterine infection, whereas the treatment with estradiol, the major female estrogen sex hormone, reduced the susceptibility to infection (151).

The sex steroid hormones are also important mediators of inflammation (152) and may influence resistance or susceptibility to parasitic infections (153). Sex steroid hormones are also involved in viral infections. It has been proposed that HIV-1 utilizes a window of vulnerability during the menstrual cycle. This crucial period overlaps with the mid-cycle when innate and adaptive immune responses are suppressed by estrogens and/or progesterone to facilitate reproductive processes. HIV-1 presumably exploits this time frame, during which antiviral factors are suppressed, to establish and propagate infection in the female mucosal genital tract (31, 148, 154). In menopausal women, the low concentration of hLf in the secretions, related to the low levels of sexual hormones (155), may lead to recurrent infections.

It is important to underline that during menses the decrease of this important natural defense glycoprotein is balanced by the presence of neutrophils that, through the synthesis of granules, can restore, at least partially, hLf concentration and its antimicrobial activity when the epithelial barrier is disrupted. The recruitment of neutrophils occurs through high levels of inflammatory biomarkers as pro-inflammatory cytokine as IL-8, IL-6 or C-reactive protein (119). As matter of fact, during menstruation, as well as in aging and menopause, the decrease of estrogens is related to the increase of inflammatory processes (156).

Interestingly, hLf expression in endometrium suggests that, during the gestational period, hLf produced by uterine epithelium and neutrophils and released in CVF is controlled by sex steroid hormones (149). It has been reported that high levels of progesterone parallel low levels of estrogens in normal pregnancy, while this ratio is inverted in pregnant women with the preterm delivery threat (157). Determining the existence of a regulatory circuit linking hLF synthesis and sex steroid hormone fluctuations may unravel novel mechanisms leading to preterm birth.

## Conclusion

*Lactobacillus* spp. and Lf are pivotal components of first-line defense in the female mucosal genital tract involved in protection against a multitude of microbial infections and the most effective natural mechanism to dampen inflammatory processes.

To inhibit cervicovaginal infections, an ideal drug should inhibit:

- microbial growth;
- microbial adhesion and entry into host cells;
- microbial intracellular replication; and
- infection of new host cells by microbes extracellularly released from the infected cells.

In the vaginal environment of women of childbearing age, the inhibition of bacterial multiplication through the synthesis of antibacterial substances by lactobacilli or by competition between microbes and Lf for iron acquisition represents an effective natural defense mechanism. Both lactobacilli and Lf can inhibit the adhesion and consequently the microbial entry inside the cells through an interaction with the cell surface components potential receptors for pathogens. Lactobacilli and Lf appear complementary since lactobacilli inhibit microbial intracellular replication and together with Lf hinder the infection of still healthy cells by microbes extracellularly released. This close cooperation is also exerted through their anti-inflammatory function. In this scenario, the mucosal environment represents a good model of mutualism and reciprocity against the injury by microbes.

**Figure 2:**
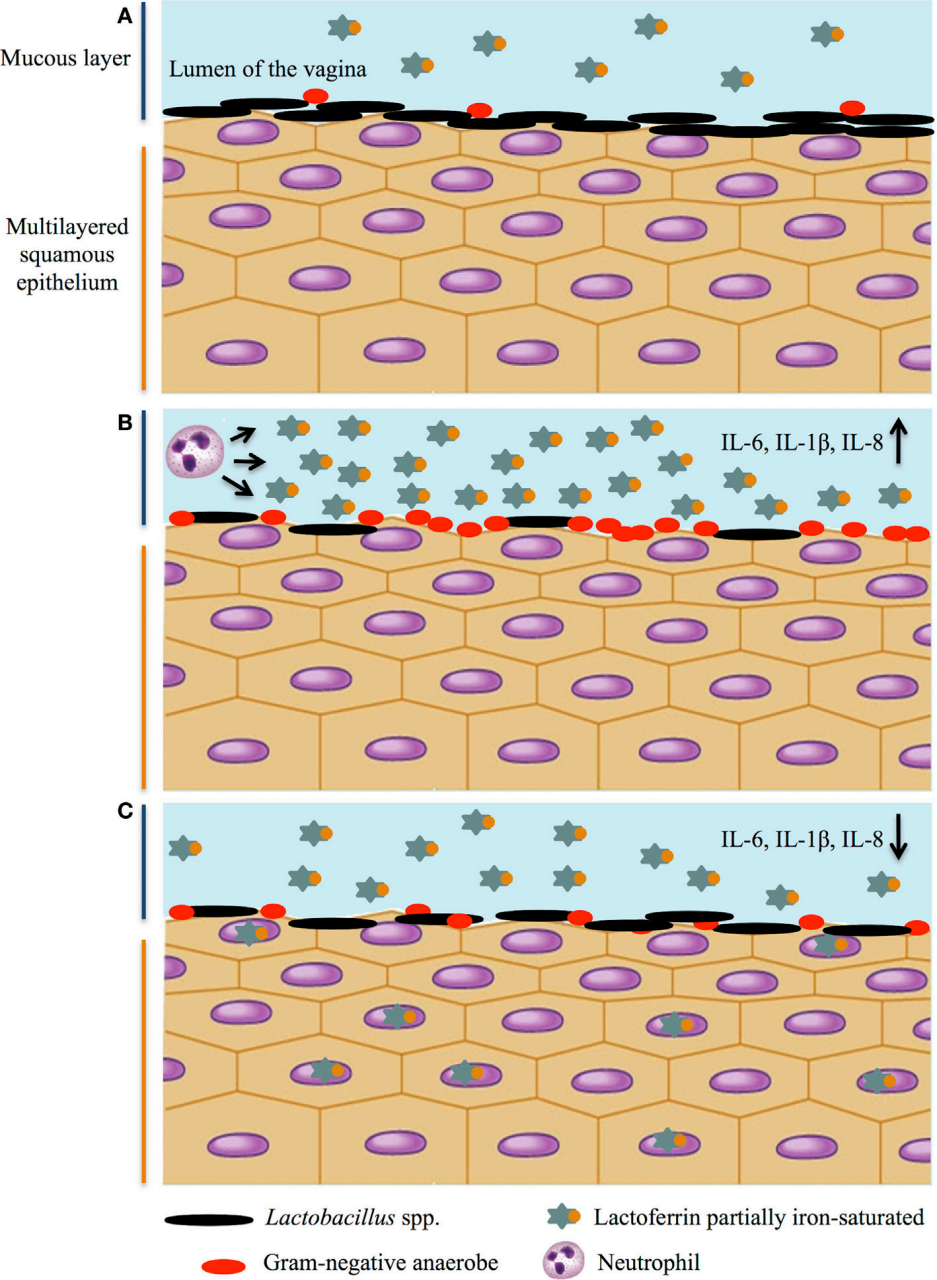
*Lactobacillus* spp. and lactoferrin interplay on infection and inflammation in female genital tract. A schematic representation of *Lactobacillus* spp. and lactoferrin balance: a multitasking strategy to protect against pathogen challenge and maintain immune homeostasis. **(A)** Healthy genital tract; **(B)** vaginosis: high levels of pro-inflammatory cytokines, decrease of lactobacilli and increase of Gram-negative anaerobes, and increase of lactoferrin concentration released by neutrophils; **(C)** decrease of pro-inflammatory cytokines by lactoferrin and restoration of healthy microbiota.

A schematic representation of *Lactobacillus* spp. and Lf balance in protecting against pathogens and maintaining immune homeostasis in the vaginal tract is shown in Figure 2.

Considering the shortage of effective treatments to counteract antibiotic-resistant bacterial infections, the intravaginal administration of lactobacilli and Lf could be a novel efficient therapeutic strategy and a valuable tool to restore mucosal immune homeostasis.

## Author Contributions

PM, RP, and PV conceived the topic concept and wrote and revised the final manuscript; LR, AC, ML, DC, and ES provided figures and contributed to manuscript preparation and editing. All authors read and approved the final version.

## Conflict of Interest Statement

The authors declare that the research was conducted in the absence of any commercial or financial relations hips that could be construed as a potential conflict of interest.
